# Nontargeted metabolomic profiling analysis of patients with type 2 diabetes mellitus undergoing corn silk treatment

**DOI:** 10.1097/MD.0000000000039396

**Published:** 2024-08-16

**Authors:** Yu Cheng, Hong Chao, Jinghua Liu, Jicheng Liu

**Affiliations:** a Postdoctoral Workstation, Research Institute of Medical and Pharmacy, Qiqihar Medical University, Qiqihar, China; b Postdoctoral Research Station, Heilongjiang University of Chinese Medicine, Harbin, China; c Department of Preventive Medicine, School of Public Health, Qiqihar Medical University, Qiqihar, China

**Keywords:** corn silk, fasting blood glucose, HbA1c, type 2 diabetes mellitus, untargeted metabolomics

## Abstract

To explore the corn silk’s effect and possible mechanism on patients with type 2 diabetes mellitus (T2DM) by untargeted metabolomics. Newly diagnosed patients with T2DM admitted to the endocrinology department of the author’s hospital from March 2020 to September 2021 were chosen and then allocated to either the intervention or the control group (NC) randomly. Patients in the intervention group were administered corn silk in the same way as the patients in the NC were given a placebo. A hypoglycemic effect was observed, and an untargeted metabolomics study was done on patients of both groups. Compared with the NC, the glycosylated hemoglobin and fasting blood glucose of patients in the intervention group significantly decreased after 3 months of treatment (*P* < .05), identified using tandem mass spectrometry, and analyzed by orthogonal partial least squares-discriminant analysis. A total of 73 differential metabolites were screened under the conditions of variable important in projection value >1.0 and *P* < .05. Differential metabolites are mainly enriched in signaling pathways such as oxidative phosphorylation, purine metabolism, and endocrine resistance. Through untargeted metabolomic analysis, it is found that corn silk water extract may reduce blood glucose in patients with T2DM through multiple pathways, including oxidative phosphorylation and purine metabolism.

## 1. Introduction

The latest data from the International Diabetes Federation revealed that the global number of adults diagnosed with diabetes has reached 537 million, a concerning statistic. In China, the incidence of type 2 diabetes mellitus (T2DM) is on the rise due to rapid economic development and dietary changes.^[1]^ Prolonged high blood glucose levels can lead to complications such as diabetic nephropathy and diabetic foot. The development of hyperglycemia in T2DM is a multifaceted process, involving various factors such as increased nonesterified fatty acids, inflammatory cytokines, adipokines, and mitochondrial dysfunction leading to insulin resistance, as well as glucotoxicity, lipotoxicity, and amyloid formation contributing to β-cell dysfunction.^[2]^ Therefore, effective blood glucose management is crucial in the treatment of T2DM. Patients with poor glycemic control may require a combination of multiple hypoglycemic drugs or intensive insulin therapy.^[3]^

Traditional Chinese medicine (TCM) exhibits advantages in the treatment of T2DM. T2DM, categorized as “Xiaoke” (means quench thirst), is one of the diseases where the endocrinology department of our hospital has an advantage in treatment over Western medicine. In TCM theory, T2DM is caused by a deficiency in origin and excess in superficiality. To be more specific, improper diet and poor congenital endowment may lead to burnout, fatigue, shortness of breath, and weakness of spirit, usually accompanied by deficiency of Yin and body fluid. If not interrupted, it will further lead to a deficiency in Qi and Yin, causing blood stasis and collateral blockage. Therefore, for patients with T2DM with poor glycemic control resulting from Qi and Yin deficiency, the treatment, adhering to the principle of treating both the symptoms and root causes, should nourish Qi and Yin, strengthen the spleen and kidney, promote blood circulation, and remove blood stasis. Corn silk is the style and stigma of gramineous maize, a crop planted in most parts of China. The earliest medicinal use of corn silk was found in the Southern Yunnan Materia Medica. Recent studies have found that corn silk decoction can alleviate insulin resistance by improving the body’s antioxidant capacity, promoting glycogen synthesis, reducing glycogen decomposition, and enhancing the sensitivity of liver and skeletal muscle to insulin.^[4,5]^

In untargeted metabolomics analysis, liquid chromatograph-mass spectrometer, gas chromatography-mass spectrometry, and nuclear magnetic resonance technologies are adopted to unbiasedly detect the dynamic changes of all small molecule metabolites before and after stimulation and perturbation in cells, tissues, organs, and organisms and followed by bioinformatics analysis to screen them. Differential metabolites, a method for pathway analysis of differential metabolites, reveal the physiological mechanisms of their changes.^[6]^ In previous research,^[7,8]^ we have identified the components of corn silk and found the main active ingredients in its water extract, namely, ferulic acid, caffeic acid, protocatechuic acid, vanillin, vanillic acid, salicylic acid, and celery white. At the moment, the majority of studies on the treatment of T2DM using an aqueous extract of corn silk are based on experiments conducted on animals. Therefore, this study plans to investigate the mechanism of water extract of corn silk in the treatment of T2DM using untargeted metabolomics, with the goal of providing additional references for the therapeutic treatment of patients with T2DM with early diagnosis.

## 2. Materials and methods

### 2.1. Samples

Newly diagnosed patients with T2DM admitted to the endocrinology department of the author’s hospital from March 2020 to September 2021 were selected and then divided into 2 groups, namely, the corn silk intervention group (CS) and the control group (NC), by random number grouping. We searched for some relevant research results to estimate the sample size. In an earlier clinical study conducted in China, the mean fasting blood glucose (FBG) levels were 7.92 mmol/L in the NC and 7.26 mmol/L in the CS, with a standard deviation of 0.7 mmol/L. Assuming that in the bilateral significance test with a power of 0.8 and an alpha error level of 0.05, we have used the sample size calculation formula $N=2{\left[\frac{\left(Z_{\alpha {/}_{2}}+Z_{\beta }\right)\sigma }{\left(\mu_{2}-\mu_{1}\right)}\right]}^{2}$ to estimate the required sample size for our study. At least 18 patients were required in each group to compare this change between the groups with 80% power using a *t* test. The randomization was carried out through an online web-based tool (freely available at http://www.randomizer.org/). For concealment of allocation, the randomization procedures and assignment were managed by an independent research assistant who was not involved in the screening or evaluation of the participants. All patients were informed of the study and required to sign the informed consent before inclusion. The research project was approved by the Ethics Committee of Qiqihar Medical College (2020) 24. All procedures comply with the ethical guidelines of the Declaration of Helsinki on clinical research.

### 2.2. Inclusion and exclusion criteria

#### 2.2.1. Inclusion criteria

Aged from 18 to 80 years old; met the diagnostic criteria for T2DM issued by the Chinese Guidelines for the Prevention and Treatment of Type 2 Diabetes (2020 Edition)^[9]^; newly diagnosed with T2DM and received no relevant treatment before inclusion; in normal psychiatric status; and agreed to participate in the study voluntarily and signed informed consent.

#### 2.2.2. Exclusion criteria

With complications of diabetes; with allergic constitution or history of TCM allergy; pregnant or in breastfeeding period; with insufficiency of heart, liver, kidney, and other important organs; with other chronic or infectious diseases; suffered from serious complications such as hyperosmolar and ketoacidosis in the past month; with malignant tumors; with autoimmune diseases; and allergic or intolerant to the drug in this study.

### 2.3. Interventions

Patients in the intervention group took 4.8 g of test capsules (CSAE) per day according to the recommended dosage in the Pharmacopoeia. CSAE, supervised by the pharmacy department of the hospital, is the water extract from about 60 g of dry corn silk. Twice a day, CSAE is taken 30 minutes before breakfast and dinner with warm water. Patients in the NC were given a placebo, which was administered in the same way as that in the intervention group. At the same time, the 2 groups of patients received basic interventions according to the Chinese Guidelines for the Prevention and Treatment of Type 2 Diabetes (2020 Edition) for a period of 3 months.^[9]^

### 2.4. Observation of hypoglycemic effect

Before and after the 3-month treatment, fasting and 2-hour postprandial venous blood were collected from all patients for laboratory testing to determine the levels of FBG, glycosylated hemoglobin (HbA1c), and C-peptide.

### 2.5. Untargeted metabolomics

Collected blood samples were centrifuged at 4000 rpm for 15 minutes at 4 °C. After being separated, the serum was added dropwise to 40 μL of phosphoric acid and then vortexed for 60 s. Oasis HLB SPE cartridges were preactivated with 2-mL methanol and 2-mL water, respectively. The resulting mixed solution was added to the column. With effluent discarded, 2 mL of 100% methanol was used to elute. The eluate was collected and then dried under nitrogen flow at 4 °C. Dried residue was redissolved in 100 μL of 100% methanol and vortexed for 60 s. The solution was centrifuged at 13,000 rpm for 15 minutes at 4 °C, filtered with a 0.22-µm filter, and was injected 3 μL of reagents for UHPLC-Q-TOF-tandem mass spectrometry (MS/MS) analysis.^[7]^

### 2.6. Statistical processing

SPSS 20.0 software was used for statistical analysis of the data. Measurement data conforming to normal distribution were expressed as mean ± standard deviation $\left(n=\frac{2{\left(\mu_{\alpha }+\mu_{\beta }\right)}^{2}p(1-p)}{\delta^{2}}\right)$, and an independent sample *t* test would be performed if homogeneity of variance is reached; otherwise, an independent sample *t*′ test would be performed. A paired sample *t* test was applied for pretest and posttest comparison within the group. Enumeration data were expressed by frequency or constituent ratio and verified using the χ^2^ test. *P* < .05 means that the difference is statistically significant.

The data collected by UHPLC-Q-TOF-MS/MS were imported into Progenisis QI software for peak matching and peak extraction and then imported into EZinfo software. Principal component analysis and partial least squares discriminant analysis (orthogonal partial least squares) were performed on each group of data. Differential metabolites were picked out when important projected value (variable important in projection) >1, *P* < .05 in *t* test, and fold change >2.

The variation trend of the differential metabolites and the receiver operating characteristic (ROC) curve were observed by cluster analysis to further verify the differential metabolites. Database (KEGG/HMDB) was consulted for the structural identification of metabolites, and corresponding metabolic pathways were identified to explore the biological significance of potential biomarkers on the basis of relevant literature and molecular biology knowledge.

## 3. Results

### 3.1. Basic information of selected patients

Thirty-six patients with T2DM were recruited in the trial, 18 in the CS and 18 in the NC, aged between 30 and 68 years, with an average age of (60.39 ± 12.24) years and an average body mass index of (27.13 ± 3.67) kg/m^2^; among them, 13 were males and 23 females. There was no significant difference in overall conditions between the 2 groups of patients (Table 1).

**Table 1:** Basic information of selected patients.

	NC (N = 18)	CS (N = 18)
Age, yr; mean (SD)	61.4 (11.3)	59.9 (10.5)
Female, n (%)	6 (33.3)	7 (38.9)
Weight, kg; mean (SD)	86.7 ± 16.1	85.1 ± 17.4
BMI, kg/m^2^; mean (SD)	36.7 (3.3)	28.9 (3.8)
FBG, mmol/L; mean (SD)	9.3 (2.6)	9.2 (2.7)
HbA1c, %; mean (SD)	8.23 (0.81)	8.15 (0.79)
C-peptide, ng/mL; mean (SD)	2.45 (0.92)	2.49 (0.97)

N is the number of patients in the full analysis set.

BMI = body mass index, CS = corn silk intervention group, FBG = fasting blood glucose, HbA1c = glycosylated hemoglobin, NC = control group.

### 3.2. Hypoglycemic effect

**Figure 1.**
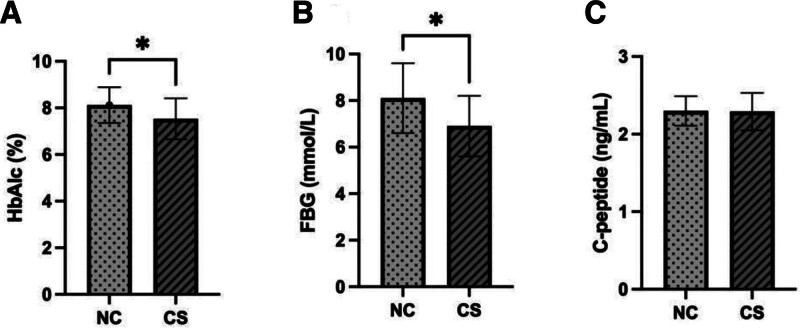
Comparison of hypoglycemic effect between 2 groups. FBG = fasting blood glucose, HbA1c = glycosylated hemoglobin.

After 3 months of therapy, the HbA1c and FBG levels of patients in the CS were considerably lower than those in the NC (*P* < .05; Fig. 1A and B). There was no significant difference in serum C-peptide concentration between the 2 groups (Fig. 1C).

### 3.3. Untargeted metabolomics

#### 3.3.1. Screening of differential metabolites

**Figure 2.**
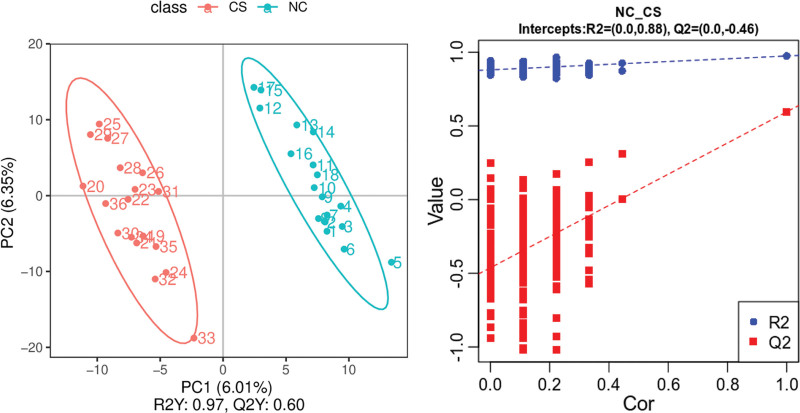
OPLS-DA map of 2 groups of patients. CS = corn silk intervention group, NC = control group, OPLS-DA = orthogonal partial least squares-discriminant analysis, PC = principal component.

At the end of the treatment, the processed samples were detected, and a total of 447 metabolites were identified by MS/MS and analyzed by orthogonal partial least squares-discriminant analysis and permutation test (Fig. 2). The model coefficient R^2^Y of the 2 groups is 0.97, indicating that the model has high predictive ability and good fitting degree. In the permutation test, the Q^2^ points of the model are far lower than the original Q^2^ points at the rightmost end, R^2^ = 0.88, and the intercept of the Q^2^ regression line is −0.46, indicating that the model has good predictive ability and can be effectively used. A total of 73 differential metabolites were screened under the conditions that variable important in projection value >1.0 and *P* < .05.

#### 3.3.2. Cluster analysis

**Figure 3.**
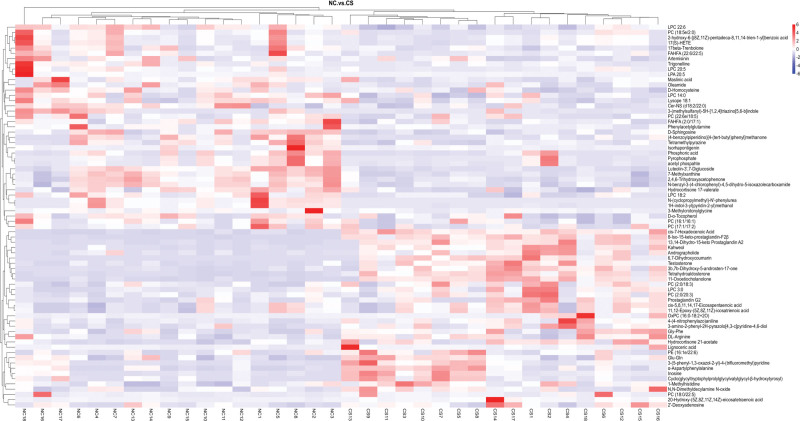
Metabolite clustering heatmap of control and intervention group. CS = corn silk intervention group, NC = control group.

As illustrated in Figure 3, cluster analysis was employed to graphically demonstrate the variation trend of differential metabolites. Sample groups and possible biomarkers are shown on the horizontal and vertical axes, respectively. In the figure, red denotes the highest level, blue represents the smallest amount, and the names of differential metabolites are marked on the vertical axis on the right. The findings showed substantial variations in metabolites between the control and the intervention group.

#### 3.3.3. ROC curve analysis

**Figure 4.**
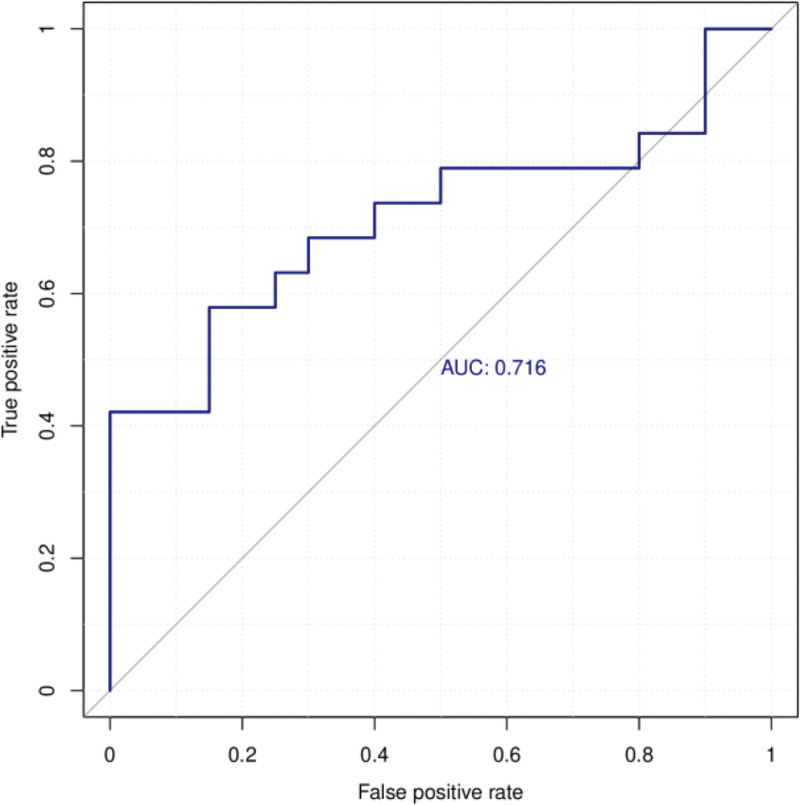
Receiver operating characteristic curve analysis of heterosexual metabolites. AUC = area under the curve.

Multiplex exploratory ROC curve analysis was adopted to assess the sensitivity and specificity of various metabolites (Fig. 4). The area under the curve of the differential metabolites in the 2 groups was 0.716, confirming that the screened differential metabolites exhibit high sensitivity and specificity.

#### 3.3.4. Pathway enrichment analysis

**Figure 5.**
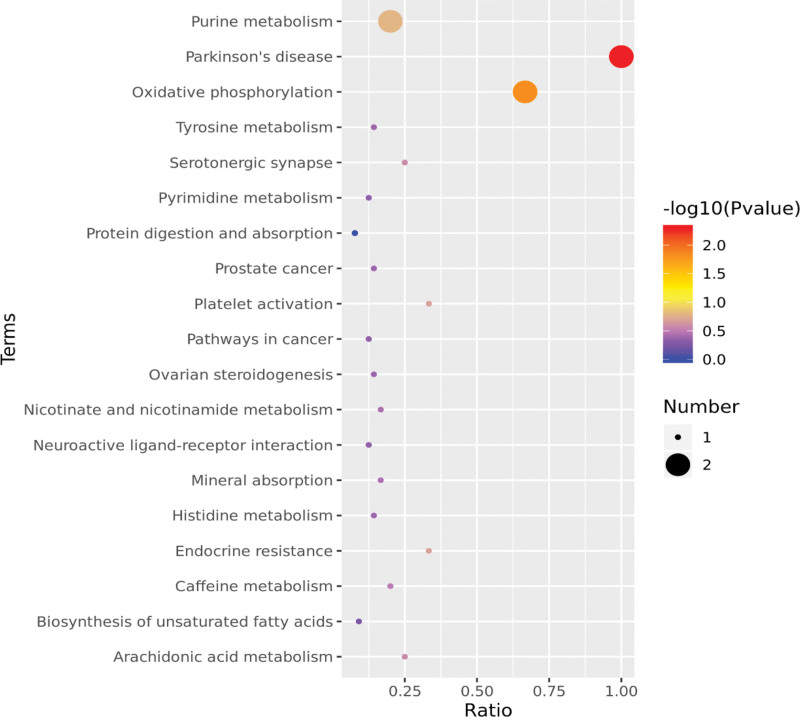
Pathway enrichment analysis of differential metabolites.

For metabolic pathway analysis, the observed differential metabolites were imported into the HMDB and KEGG databases. According to the findings, those metabolites were primarily enriched in oxidative phosphorylation, purine metabolism, endocrine resistance, Parkinson pathway, and other signaling pathways, demonstrating that patients in the intervention group experienced significant changes in their body’s energy metabolism. As illustrated in Figure 5.

## 4. Discussion

In the 21^st^ century, with the rapid development of the economy, continuous increase in the pressure in work and life, and the explosive growth of material resources, the traditional regular life routine of “work at sunrise and rest at sunset” was replaced by diversified urban lifestyles with diet increasingly shift to food of high fat and high calories. As a result, the proportion of fat and obese population becomes increasingly higher year by year and so is the incidence of metabolic diseases. The incidence of diabetes, a typical metabolic disease, has seen a sharp increase in recent years, which has affected the physical and mental health of a wider range of populations and brought a heavy burden to families with diabetic patients and society. More than 90% of diabetic patients were diagnosed with T2DM, and among them, 75% to 80% were obese with T2DM.^[10–12]^ Currently, in the treatment of an abdominal obese type of T2DM, in addition to the use of conventional oral hypoglycemic drugs, modern medicine focuses on accelerating the metabolism and decomposition of energy substances, such as sugar and lipids. However, drugs of the abovementioned functions are so expensive, which are not affordable for most patients in the long term. Besides, many patients cannot resist their stronger-than-normal appetite and thirst for food of high sugar and fat; some patients even present overeating behaviors, which increases the difficulty of treatment and decreases the effect of treatment. Therefore, the key to treatment mainly lies with the patients themselves.^[13–15]^

In TCM, diabetes belongs to the category of “Xiaoke (quench thirst).” Diabetes is considered to be related to congenital factors, improper diet, imbalance of work and rest, exogenous 6 evils, internal injury, and emotions.^[16]^ Therefore, TCM syndrome differentiation treatment regulates the stomach, lung, and kidney, adhering to the principle of strengthening the spleen and nourishing Qi, clearing heat and nourishing Yin, clearing dryness and heat, and restoring Yin deficiency. China is a corn-growing country. Back in ancient times, TCM practitioners adopted corn silk in the prescription for diabetes.^[17]^ According to Chinese Pharmacopoeia, corn silk has a play not only along the Yangming meridian of the stomach but also meridians of the kidney, liver, and gallbladder meridians, with the effect of reducing swelling and clearing heat of the liver and gallbladder.^[18]^ In recent years, with the development of molecular and cell biology, the effect of corn silk in lowering blood glucose, blood lipid, and even cholesterol levels has been further confirmed.^[19,20]^

Besides, pharmaceutical research also found that both corn silk saponins and corn silk polysaccharides play a certain role in lowering blood lipids, with the former playing the main role.^[21]^ Corn silk, with ingredients such as sterol, flavonoids, alkaloids, polysaccharides, and others, is inductive to clearing dampness and water, removing jaundice, and reducing swelling. Modern pharmacology found that corn silk is effective in lowering blood glucose and lipids, which lays the ground for its use in the treatment and prevention of metabolic syndromes.^[22]^ An animal experiment showed that the water extract of corn silk has the effect of lowering blood glucose levels in T2DM mice induced by streptozotocin.^[23]^ Another report stated that corn silk ethanol extract not only exerts an obvious hypoglycemic effect but also has antioxidant activity, which can effectively prevent the occurrence of diabetic nephropathy.^[24]^

Results of the study found that compared with the effect seen in the NC, after 3 months of treatment, corn silk could further lower the FBG and HbA1c levels of the patients in the intervention group. In a previous study, we used UHPLC-Q-TOF-MS/MS technology to identify the components of water extract of corn silk and found 76 compounds, including caffeic acid and 10 derivatives thereof, (E)-p-coumaric acid and 2 its derivative ingredients, ferulic acid and 4 kinds of its derived ingredients, and 5 kinds of flavonoids. In this study, further analysis of the chemical components in the water extract of corn silk was conducted, and 21 components were initially identified, including 9 prototype components and 12 blood metabolites.^[7,8]^ Therefore, it is believed that the active ingredients in the water extract of corn silk are mainly ferulic acid, caffeic acid, metabolite acid of protocatechuic acid, vanillin, vanillic acid, salicylic acid, apigenin, and the prototype aldehyde of vanillin.

T2DM can cause abnormal metabolism of many substances in the body, such as carbohydrates, lipids, and amino acids. An increasing number of research results suggest that branched-chain amino acids (BCAAs), including valine, leucine, and isoleucine, are possible biomarkers for T2DM. Insulin resistance was found to be linked with higher plasma levels of BCAAs, aromatic amino acids, and higher glutamate-to-glutamine ratios in Singapore research.^[25]^ This study showed that the metabolism of Gly-Phe, 1-methylhistidine, α-aspartylphenylalanine, 3-methylcrotonylglycine, DL-arginine, D-methionine, and Glu-Gln changed in patients of the intervention group after the treatment with corn silk. Changes in the metabolism of amino acids may be due to the therapeutic effects of corn silk. A study by Pedersen et al^[26]^ integrated data on subjects’ insulin sensitivity, gut microbiome, and fasting serum metabolome and found that the serum metabolomic signature of insulin-resistant individuals was elevated BCAA levels. Patients with T2DM often experience dyslipidemia. Lu et al^[27]^ conducted liquid chromatograph-mass spectrometer and gas chromatography-mass spectrometry untargeted metabolomic studies on the serum of Chinese people and found that some free fatty acids (palmitic acid, stearic acid, oleic acid, and linoleic acid) in patients with T2DM were significantly higher. Similar findings were also spotted in this study. The metabolism of fatty acids such as eicosatetraenoic acid, eicosapentaenoic acid, and eicosatrienoic acid changed in patients of the intervention group.

Enrichment analysis on the signaling pathways of different metabolites was then performed in this study, and the findings revealed that differential metabolites were mostly enriched in signaling pathways such as oxidative phosphorylation, purine metabolism, and endocrine resistance. More and more fundamental and clinical research has revealed that oxidative stress is directly linked to the pathological alterations associated with diabetes. Hyperglycemia increases the generation of reactive oxygen species due to hyperactivity of polyol metabolism, auto-oxidation of glucose, and oxidative phosphorylation. The nonenzymatic glycation reaction reduces the antioxidant capacity of SOD, triggers a state of oxidative stress, damages biological macromolecules, changes intracellular information transmission, and damages the structure and function of cells. It is known to all that uric acid is the end product of purine metabolism. The occurrence of T2DM and hyperuricemia share many common pathogenic bases, so patients with T2DM are more likely to have hyperuricemia. The increase in the uric acid caused by purine metabolism can also affect islet function, increase insulin resistance, and so make it more difficult to control blood glucose in diabetic patients. Therefore, it is inducted from our research results that corn silk is likely to treat patients with T2DM through multiple mechanisms such as oxidative phosphorylation pathway and purine metabolism pathway. The last thing worth pointing out is the Parkinson pathway. Studies have found that there are many interpretable links between Parkinson disease and T2DM.^[28]^ Genetics may play a key role in younger patients, while in older patients, the degenerative process itself may disrupt brain-driven endocrine pathways and associated pathways. Therefore, our findings also suggested that T2DM may be improved by modulating the Parkinson pathway.

As for limitations of the study, a small sample size may increase the error of results; results of the untargeted metabolomic analysis were not further validated, so some conclusions can only be inducted theoretically. In addition, the study period is relatively short, during which irregularities may occur in the treatment of patients and affect the results.

## 5. Conclusion

Through untargeted metabolomic analysis, this research showed that corn silk may lower blood glucose in patients with T2DM through multiple pathways such as oxidative phosphorylation and purine metabolism. Based on the TCM theory, this research administered drugs of concrete dose from standard capsule units instead of conventional TCM compounds, in the hope of providing a new direction for clinical TCM treatment of T2DM. We hope to see future research in this direction and wider use of corn silk in clinical treatment.

## Acknowledgments

The authors are very grateful to the doctors and nurses in the participating hospitals for their help with collecting the data and patients’ samples. They would also like to thank all the study staff and participants for their contribution.

## Author contributions

**Conceptualization:** Yu Cheng, Hong Chao, Jinghua Liu, Jicheng Liu.

**Funding acquisition:** Yu Cheng.

**Investigation:** Yu Cheng, Hong Chao, Jinghua Liu.

**Writing – original draft:** Yu Cheng, Hong Chao.

**Writing – review & editing:** Yu Cheng, Jicheng Liu.

**Formal analysis:** Hong Chao, Jinghua Liu.

**Supervision:** Jinghua Liu.

**Methodology:** Jicheng Liu.
